# Maternity and Child Welfare

**Published:** 1918-08-31

**Authors:** 


### The Hospital

The Workers' Newspaper of Administrative Medicine and Institutional Life, Administration, National Insurance and Health.

No. 1682, Vol. LXIV. SATURDAY, AUGUST 31, 1918. PRICE TWOPENCE

### MATERNITY AND CHILD WELFARE.

The pessimistic school of philosophy, which teaches that human life is so full of misery, so futile in its achievements, so uncertain of its goal, that it were best ended by abstaining from renewing it, has few disciples amongst men of British stock. The assumption of the responsibility of perpetuating the race, however, should carry with it the duty of ensuring the health and well-being of the new generation. And this is not only an individual but a communal duty, because the health of a State is but the sum total of the health of all its members, and no State can continue to flourish if the children of its citizens fail. The State of the future will probably be as painstaking in the choice of parents as the magistrates of Plato's "Republic;" but the advocate of eugenic principles has much educational work to do before he can hope to see his views enforced. Until our knowledge of the laws of heredity is much greater and more certain we cannot hope to control the breeding of the race by legislative enactments. There is, however, general agreement that the expectant mother should be shielded as far as possible from the dangers of pregnancy and parturition, and every opportunity given her to produce and rear a healthy child.

From this point of view the Maternity and Child Welfare Act of 1918 is a very useful addition to our public health legislation. The Local Government Board has recently issued to County Councils and Sanitary Authorities a circular* explaining the Act and suggesting methods of putting it into force. The circular points out that local authorities can now receive grants for the provision of hospital treatment for children up to five years of age; of lying-in and convalescent homes, day nurseries, and homes for the children of widowed, deserted, and unmarried mothers; of food for expectant and nursing mothers and for children under five, and of home helps during lying-in; and for the promotion of experimental work bearing on the health of mothers and children. The Board hopes that each County Council will draw up for the whole county a comprehensive scheme, which will link up the work done by all the local authorities within the county area. Such a scheme should provide for the adequate provision and supervision of midwives, Health Visitors, nurses, and home helps, for the formation of Maternity and Child Welfare Centres, and for sufficient hospital accommodation. It is suggested that home-nursing services should be organised for expectant mothers, maternity nursing, the nursing of measles, whooping cough, epidemic diarrhœa, ophthalmia neonatorum, and puerperal fever. If closely supervised by medical practitioners this home nursing will be a valuable means of reducing the call for hospital accommodation. Home helps are to be provided when it is desirable to remove the woman to a hospital for her confinement, either on account of unsuitable home conditions or of abnormal pregnancy. These home helps are to undertake such ordinary domestic duties as cleaning, cooking, washing, care of children, mending, and marketing. It is obvious that great care will be needed in choosing them, and that they must be properly trained in their work. If the right kind of woman is obtained her value will soon be appreciated in the district in which she practises, and the natural unwillingness many women show at present to leave their homes to go to a hospital for confinement will be overcome.

One great difficulty in carrying out the Act to the greatest advantage is the shortage of medical men. The instruction of Health Visitors and the work of the Centres should be under their close supervision. But medical men are too busily employed to undertake the work, and the circular therefore refrains from laying down any special course of training for Health Visitors, and suggests merely that every effort should be made to secure the attendance of a doctor at a Centre at least once a fortnight. The Cheshire doctors have taken alarm at this, and have drawn the attention of the President of the Local Government Board to the evils likely to arise from the teachings of partially trained or untrained workers of approved Child Welfare Centres. The alternative, however, would be the hanging up of the whole scheme until the end of the war. The better way would seem to be, as the Board proposes, to make use of the means to hand and to develop them as opportunity arises.

* Summarised on page 480.

The Board trusts that all Local Authorities will arrange to pay the fees of doctors called in by the midwives, but it does not, as it should have done, make the payment of a grant depend upon the adoption of this rule.

Next to the want of sufficient medical men the greatest obstacle to utilising the Act to the greatest advantage will be the lack of sufficient hospital accommodation. The Mansion House Council on Health and Housing in its report just published says "there is an insufficiency in most districts for the institutional treatment of infants and young children. Except in a few instances this deficiency cannot be made good by the existing hospitals save at the expense of the older children." The report deals only with London, but the conditions in the rest of the country are still worse. The Local Government Board does not mention the Poor-Law infirmary in its circular. It implies that the whole of the hospital accommodation required is to be sought for in the voluntary hospitals. The reason is obvious. The understaffing, both medical and nursing, of the Poor-Law infirmary—more obvious in the children's wards than in any other department—and the stigma attaching to its connection with the Poor Law, would be insuperable bars to its use. Yet we have in the Poor-Law infirmaries, if properly managed and organised, accommodation amply sufficient to meet all demands. How long must we wait before our legislators will free the Poor-Law infirmaries from their connection with the Poor Law, and bring them into relation with the new schemes of medical treatment for the whole community?

